# Optogenetic control of transgene expression in *Marchantia polymorpha*

**DOI:** 10.1002/aps3.11632

**Published:** 2025-01-28

**Authors:** Anya Lillemor Lindström Battle, Lee James Sweetlove

**Affiliations:** ^1^ Department of Biology University of Oxford South Parks Road Oxford OX1 3RB United Kingdom

**Keywords:** acetyl‐CoA, liverwort, metabolic engineering, poly‐3‐hydroxybutyrate (PHB), toolbox

## Abstract

**Premise:**

The model liverwort *Marchantia polymorpha* is an emerging testbed species for plant metabolic engineering but lacks well‐characterized inducible promoters, which are necessary to minimize biochemical and physiological disruption when over‐accumulating target products. Here, we demonstrate the functionality of the light‐inducible plant‐usable light‐switch elements (PULSE) optogenetic system in *Marchantia* and exemplify its use through the light‐inducible overproduction of the bioplastic poly‐3‐hydroxybutyrate (PHB).

**Methods:**

The PULSE system was used to drive expression of luciferase as a reporter and characterize its induction in transgenic *M. polymorpha*. Additionally, PULSE was used to drive expression of the PHB biosynthetic pathway; the accumulation of PHB under light‐inducible control was compared to constitutive overexpression.

**Results:**

PULSE was fully functional and minimally leaky in *M. polymorpha*. The presence of the PULSE construct, even in the absence of induction, resulted in a developmental phenotype. Constitutive and inducible expression resulted in similar PHB accumulation levels.

**Discussion:**

PHB biosynthesis in plants is known to adversely affect plant health, but placing its production under optogenetic control alleviated negative effects on biomass accumulation in some instances. The work presented here represents a significant expansion of the toolbox for the metabolic engineering of *M. polymorpha*.

Inducible promoters are invaluable in plant metabolic engineering, addressing challenges posed by product accumulation and metabolic disruption. These include promoters activated by small molecules such as ethanol, estrogen, or auxin (Corrado and Karali, 2009). However, chemically induced systems depend on chemical diffusion into tissues, organs, and cells, limiting spatial and temporal control of expression. Additionally, chemical inducers can be expensive and interfere with normal cellular activities due to chemical crosstalk or toxicity (Larsen et al., 2023). Optogenetics, using light to control gene expression, avoids these issues and allows more precise spatiotemporal control over gene expression (Ochoa‐Fernandez et al., 2020). Although widely used in mammalian cell biology, its use in plant science has been limited due to the difficulties imposed by the requirement of broad‐spectrum light for plant growth. Most of the optogenetic tools adapted or developed for plants are incompatible with the growth of plants under white light because the specific wavelength of light used for induction will fall within the broad spectrum of white light needed for normal plant growth (Shikata and Denninger, 2022).

The plant‐usable light‐switch elements (PULSE) system enables plant growth without promoter activation in normal white light conditions by combining a red‐light inducible module with a repressor module that restricts gene expression to monochromatic red light. The PULSE system is fully functional in *Arabidopsis thaliana* (L.) Heynh. protoplasts, transiently infiltrated *Nicotiana benthamiana* Domin leaves, and in stable *A. thaliana* lines (Ochoa‐Fernandez et al., 2020).

The recent development of tools and techniques for the genetic engineering of the bryophyte *Marchantia polymorpha* L. (Ishizaki et al., 2008, 2015; Kubota et al., 2013; Sugano et al., 2014; Tsuboyama‐Tanaka and Kodama, 2015; Tsuboyama and Kodama, 2018; Sauret‐Gueto et al., 2020) suggests its use as a testbed species for metabolic engineering (Boehm et al., 2017; Sauret‐Gueto et al., 2020; Tse et al., 2024). Unlike most land plants, the haploid gametophyte generation is the dominant stage in bryophytes, allowing for the faster generation of homozygous transgenics. Moreover, *M. polymorpha* can be easily maintained in axenic culture using standardized cultivation conditions and possesses a unique regeneration capacity from fragments or single cells, allowing for easy and rapid propagation (Horn et al., 2021). Importantly, primary metabolism is highly conserved in the green lineage (Cannell et al., 2020), meaning that engineering interventions tested in *M. polymorpha* should be translatable to higher plants. Testing these engineering interventions in a stably transformed plant, as opposed to the commonly used transient expression in *N. benthamiana*, would also allow the study of long‐term expression or systemic effects on plant health such as growth.

In this study, we extended the application of the PULSE system to *M. polymorpha*, augmenting its metabolic engineering toolbox with optogenetic control. To date, the choice of inducible promoters for *M. polymorpha* is limited to a heat‐shock‐responsive promoter (Nishihama et al., 2016), the estrogen‐inducible XVE system (Flores‐Sandoval et al., 2016), or an auxin‐inducible promoter (Ishizaki et al., 2012). To demonstrate the utility of PULSE in *M. polymorpha*, we assessed its ability to regulate the expression of the heterologous pathway for poly‐3‐hydroxybutyrate (PHB) production, a biodegradable polymer synthesized by various eubacteria as a carbon storage compound (Mortimer, 2019). In *Ralstonia eutropha*, PHB biosynthesis uses a three‐enzyme pathway involving β‐ketothiolase, acetoacetyl‐CoA reductase, and PHB synthase to convert acetyl‐CoA to PHB (Lu et al., 2020).

Given its potential as a biodegradable polyester, the engineering of plants for PHB production has been an active research field for more than three decades (Poirier et al., 1992). However, the production of high levels of PHB is invariably associated with detrimental effects on plant health, including severe stunting, chlorosis, and loss of fertility (Poirier et al., 1995; Bohmert et al., 2000; Bohmert‐Tatarev et al., 2011; McQualter et al., 2015). It is widely hypothesized that these effects are due to the competition between endogenous metabolism and PHB production for acetyl‐CoA, a key metabolite in the central metabolism of all organisms connecting catabolic and anabolic metabolism (Oliver et al., 2009). An inducible promoter could be used to reduce these effects and avoid competition between PHB and endogenous metabolism by inducing expression at a specific point in the plant life cycle. Small‐molecule chemical induction has been used with some success for this purpose in other plant hosts (Bohmert et al., 2002; Lossl et al., 2005; Kourtz et al., 2007) but suffers from the drawbacks already mentioned. Here, we demonstrate the effectiveness of optogenetic control over PHB production, offering insights into PHB yield optimization and plant health.

## METHODS

### Plant materials and growth

Male and female *M. polymorpha* accessions (Takaragaike‐1 [Tak1] and Takaragaike‐2 [Tak2], respectively) (Ishizaki et al., 2008) were grown aseptically on one‐half strength Gamborg B5 media (0.158% [w/v] Gamborg B5 + vitamins [Duchefa Biochemie, Haarlem, the Netherlands], 2.34 mM MES monohydrate, 29.2 mM sucrose, 1.4% [w/v] agar, pH 5.6). Plants were grown in a controlled‐environment room with a 16:8 h light:dark cycle at 18/22°C day/night temperature with 10 μmol photons·m^−2^·s^−1^ supplemental far‐red light (see light spectra in Figure S1, see Supporting Information). Light spectra and intensities were measured using an AvaSpec‐ULS2048CL‐EVO spectrometer (Avantes, Apeldoorn, the Netherlands).

### Molecular cloning and genetic construct fabrication

#### Construct design

The gene parts used in genetic construct assembly are listed in Table S1. Most gene parts were synthesized by Twist Bioscience (San Francisco, California, USA). The PULSE optogenetic system (Ochoa‐Fernandez et al., 2020) and the Gateway vectors p*Mp*GWB103 (Addgene plasmid number 68557) (Ishizaki et al., 2015) and pK7WG2 (Karimi et al., 2002) were used for expression. Plasmids were designed and assembled in silico using SnapGene (version 7.1.0; GSL Biotech, San Diego, California, USA). Synthesized gene parts were codon optimized for *M. polymorpha* using OPTIMIZER (Puigbo et al., 2007) and a codon usage table obtained from the Codon Usage Database (http://www.kazusa.or.jp/codon/).

#### Construct assembly

DNA amplification using PCR was carried out using Phusion High‐Fidelity DNA Polymerase (Thermo Fisher Scientific, Waltham, Massachusetts, USA) according to the manufacturer's instructions. Primer information is provided in Table S2. PCR fragments were assembled for transformation using the NEBuilder HiFi DNA Assembly Cloning Kit (New England Biolabs, Hitchin, United Kingdom). Constructs were assembled into the pTWIST‐Entr Gateway cloning vector, and the Gateway LR Clonase II Enzyme Mix (Thermo Fisher Scientific) was used to move the constructs into one of several Gateway expression vectors according to the manufacturer's instructions.

#### Transformation and construct validation

Cloning was undertaken using α‐select Silver Chemically Competent *Escherichia coli* cells (Meridian Bioscience, Cincinnati, Ohio, USA) using a standard heat‐shock transformation protocol. All growth steps were carried out at 30°C to account for the repetitive sequences in the PULSE construct. At least five colonies per construct were screened using diagnostic restriction digestion with an appropriate FastDigest restriction enzyme (Thermo Fisher Scientific) followed by Sanger sequencing (Source Bioscience, Nottingham, United Kingdom) and full plasmid sequencing (Plasmidsaurus, Eugene, Oregon, USA). Validated sequences were transformed into *Agrobacterium tumefaciens* strain GV3101 (pMP90) (Koncz and Schell, 1986) using a standard freeze–thaw protocol. A list of all plasmids is provided in Table S3.

### Plant transformation

*Marchantia polymorpha* spores were transformed using a published *Agrobacterium*‐mediated transformation protocol (Ishizaki et al., 2008). Hygromycin (10 μg/mL) was used for selection of transgenic plants, and cefotaxime (100 μg/mL) was used to kill any remaining *Agrobacterium* from transformation and to protect against general bacterial contamination. To generate stable lines, gemmae were collected from a single gemma cup of an antibiotic‐resistant transformant and the plant was grown to maturation. Gemmae collected from these plants were used for experiments.

For genotyping, genomic DNA was extracted from a piece of *M. polymorpha* thallus between 4–8 wk old and measuring approximately 1 × 1 cm using the cetyltrimethylammonium bromide (CTAB) method (Porebski et al., 1997). Extracted genomic DNA was diluted to 20–30 ng/μL and used as a template in a standard PCR reaction (see genotyping primers in Table S2).

### Plant image acquisition

High‐resolution images of individual plants were taken using a Stemi 508 stereomicroscope (Zeiss, Oberkochen, Germany). The staining of PHB granules using Nile Blue A was based on Poirier et al. (1992). Stained *M. polymorpha* gemmae were dry mounted and imaged using a Leica SP5 confocal microscope (Leica Microsystems, Wetzlar, Germany). Images were captured using an HC PL APO 20× water immersion objective lens or an HC PL APO CS2 63× water immersion objective lens (both Leica Microsystems) at 512 × 512‐pixel resolution with 4‐line averaging. An excitation wavelength of 633 nm was used with emission collected at 650–700 nm.

### Protein purification and analysis

#### Luciferase analysis

Luciferase expression dynamics were determined using a NightSHADE photon‐counting camera (Berthold Technologies, Bad Wildbad, Germany) in the presence of exogenous d‐luciferin solution (1 mM d‐luciferin, 0.01% [v/v] Triton X‐100), which was added to the top of the plant immediately prior to the start of the experiment. Illumination with red (660 nm), blue (470 nm), far‐red (730 nm), and/or white (equal combination red, blue, and far‐red) light and image acquisition (exposure time of 300 s) were automated using the NightSHADE camera. All light intensities were 10 μmol photons·m^−2^·s^−1^.

Luciferase activity was quantified using the Dual‐Glo Luciferase Assay System (Promega, Madison, Wisconsin, USA) according to the manufacturer's instructions and a GloMax Plate Reader (Promega). A standard curve using pure firefly luciferase standard (Promega) was used to determine micrograms of luciferase in each sample.

#### Protein purification and western blotting

For soluble protein purification, thallus fragments from 4–8‐wk‐old *M. polymorpha* were flash frozen and homogenized in a TissueLyser Reaction Tube Holder cooled to −80°C using a TissueLyser II (Qiagen, Hilden, Germany) for 5 min at 25 Hz and resuspended in a 10‐fold excess (v/w) of cold grinding buffer (50 mM NaH_2_PO_4_, 150 mM NaCl, 50 mM ascorbic acid, 0.6% [w/v] PVPP‐40, 0.4% [w/v] bovine serum albumin, 5% [v/v] glycerol, 1% [v/v] TWEEN‐20, 1 mini tablet of Pierce Protease Inhibitor [Thermo Fisher Scientific], pH 8.0). The lysate was sonicated for 10 min using a Grant Ultrasonic Bath XB3 (Keison Products, Chelmsford, United Kingdom), centrifuged for 15 min at 3000 × *g* at 4°C, and the supernatant collected.

Proteins were fractionated using sodium dodecyl sulphate–polyacrylamide gel electrophoresis (SDS‐PAGE) and transferred to a nitrocellulose membrane using standard methods. Proteins containing c‐Myc and HA tags were detected with commercial antibodies (Abcam, Cambridge, United Kingdom and Merck, Darmstadt, Germany, respectively [at 1 μg/mL]). Acetoacetyl‐CoA reductase was detected using an α‐PhbB antibody (1:500 dilution) (Nawrath et al., 1994). Bound antibodies were detected using a goat α‐rabbit IgG antibody linked to horseradish peroxidase (HRP) (Merck) and the EZ‐ECL Chemiluminescence Detection Kit (Biological Industries, Kibbutz Beit‐Haemek, Israel).

### PHB quantification

The extraction of PHB was based on a published method (Bohmert et al., 2000). After derivatization with *N*‐methyl‐*N*‐(trimethylsilyl)trifluoroacetamide (MSTFA) for 30 min at room temperature with gentle shaking, a 0.25‐μL sample was injected in splitless mode at 230°C into an Intuvo 9000 gas chromatography mass spectrometer (Agilent Technologies, Santa Clara, California, USA) fitted with a 15 m × 250 μm × 0.25 μm DB‐5ms Ultra Inert Intuvo GC capillary column module (Agilent Technologies). Chromatography was performed by holding the temperature at 60°C for 5 min followed by heating at a rate of 7.5°C/min to 200°C, resulting in a run time of 24 min. An Agilent 5977B mass spectrometer (Agilent Technologies) was used in single ion monitoring (SIM) mode to detect the 189 and 203 *m/z* fragment ions for PHB and 3‐hydroxy‐valerate‐methyl ester, respectively. Samples were injected in triplicate and average values taken forward for analysis. The MassHunter MS Quantitative Analysis software (Agilent Technologies) was used for peak identification and integration with reference to the National Institute of Standards and Technology (NIST; Gaithersburg, Maryland, USA) mass spectra library (version 2.0). PHB:3‐hydroxy‐valerate‐methyl ester standard curves were generated using pure PHB standard (Merck) and used for absolute quantification.

### Data analysis and statistics

Statistics and data visualization were performed using Python (version 3.8.18, Python Software Foundation, https://www.python.org) in a Jupyter Notebook (Kluyver et al., 2016). A Mann–Whitney *U* test (Mann and Whitney, 1947) was used to determine differences between two groups and was implemented using the SciPy package (Virtanen et al., 2020). Figures were prepared using the seaborn (Waskom, 2021) and matplotlib (Hunter, 2007) packages.

## RESULTS

### Adaptation of the PULSE optogenetic system for use in *Marchantia polymorpha*

The activity of PULSE relies on the combinatorial activity of two engineered photoreceptors. The first, B_off_, represses gene expression in the presence of blue light and is built from the *Erythrobacter litoralis* EL222 photoreceptor, which binds its target DNA sequence C120_5_ as a dimer in the presence of blue light. In B_off_, EL222 is fused to the transcriptional repressor domain SRDX. The second element of PULSE is the R_on_ module, which activates gene expression in red light. R_on_ is based on the red/far‐red light‐dependent interaction between phytochrome B (PhyB) and phytochrome interacting factor 6 (PIF6) from *A. thaliana*. In PULSE, PIF6 is fused to a DNA‐binding domain that binds etr_8_, and PhyB is fused to the VP16 activator domain.

PULSE combines B_off_ and R_on_ by the construction of a synthetic target promoter, P_Opto_, which integrates the C120_5_ and etr_8_ binding sites upstream of a promoter used to drive expression of the target gene, such as luciferase. The combinatorial effect of the B_off_ and R_on_ switches ensures that blue or white light will prevent gene expression (due to binding of the repressor B_off_) and far‐red light will do the same (due to inactivity of the activator), but in red light R_on_ will bind etr_8_ and activate gene expression.

A published version of PULSE with expression of the PhyB, PIF6, and EL222 modules controlled by 35S cauliflower mosaic virus promoters (p35S), an established promoter for constitutive expression in *M. polymorpha* (Althoff et al., 2014), was used. The system was modified from its published version to carry hygromycin resistance, given the observation that *M. polymorpha* is insensitive to kanamycin (for a plasmid map, see Figure S2).

### Dynamics and distribution of PULSE expression in *Marchantia polymorpha*

**Figure 1:**
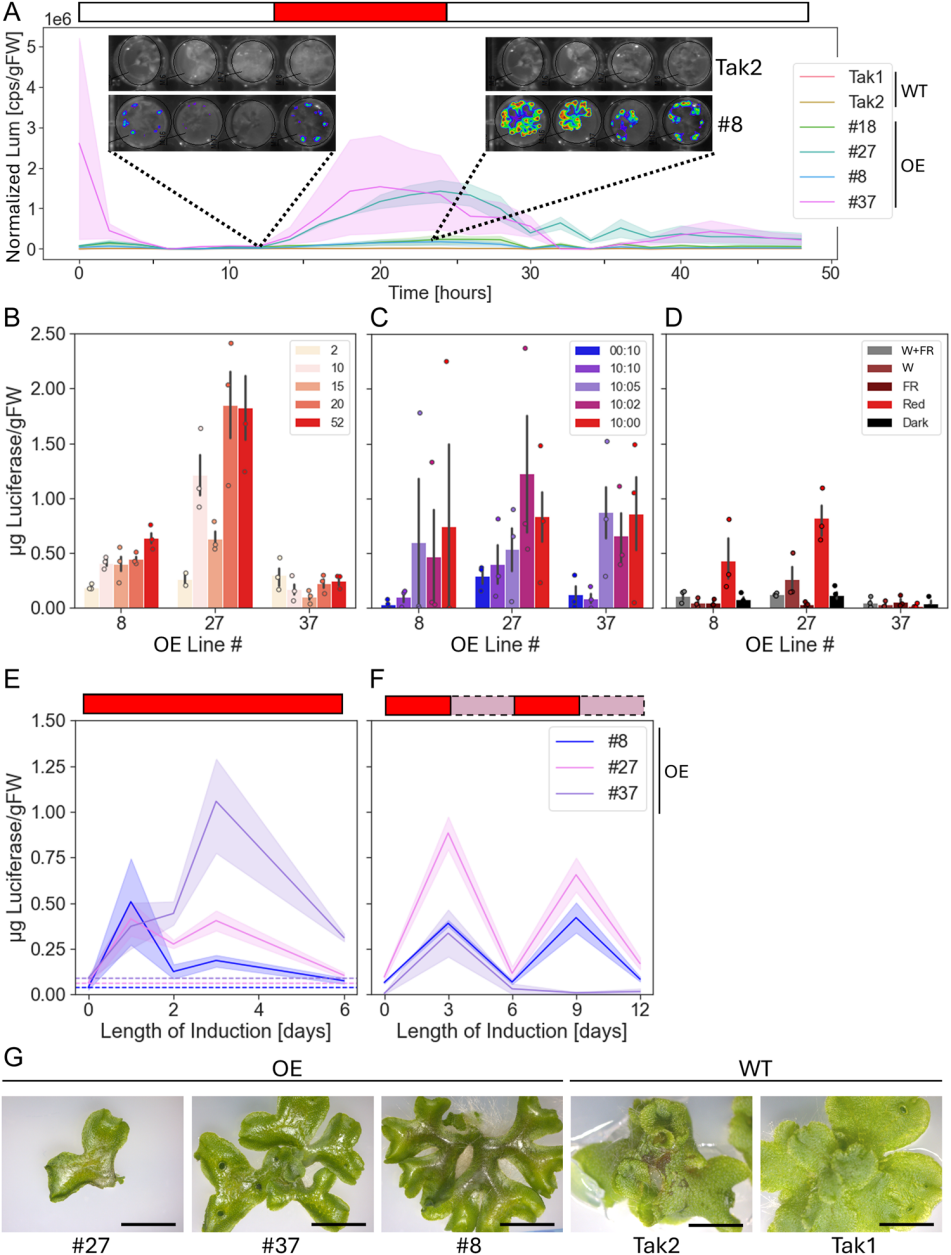
Characterization of the PULSE optogenetic system in *Marchantia polymorpha*. (A) Dynamics and distribution of expression through PULSE in *M. polymorpha*. Counts per second (cps) normalized by the final mass of each plant are shown in response to monochromatic red‐light treatment; the inlay represents raw data obtained from a photon‐counting camera, showing signal heterogeneity where red represents highest counts and blue lowest counts (*n* = 4). Shaded regions on the graph represent standard error of the mean (SEM). Bar above the figure represents the light conditions used, where red is monochromatic red light and white is white light with no supplemental far‐red light. (B) Response of the PULSE system to different intensities of red light; intensities are shown in units of μmol photons·m^−2^·s^−1^ (*n* = 3). Error bars represent SEM. (C) Response of the PULSE system to different ratios of blue to red light; ratios of blue to red light intensities are shown in units of μmol photons·m^−2^·s^−1^ (*n* = 3). Error bars represent SEM. (D) Response of the PULSE system to different white light conditions (*n* = 3). W+FR, white light with supplemental far‐red; W, white light with no supplemental far‐red; FR, far‐red. Error bars represent SEM. (E) Long‐term induction behavior of PULSE over a 6‐d period. Horizontal lines represent expression levels at *t* = 0 (*n* = 3). Shaded regions represent SEM. (F) Induction of PULSE in response to multiple three‐day pulses of red light (*n* = 3). Shaded regions represent SEM. For E and F, a bar above the figure represents the light condition used, where red is monochromatic red light and light purple is W+FR. (G) Phenotype of three PULSE lines and wild‐type *M. polymorpha* Tak1 and Tak2 plants after 25 d of growth from gemmae in W+FR conditions. Scale bar is 5 mm. gFW, gram fresh weight; OE, overexpressor/transgenic lines; WT, wild‐type.

Fifty‐five PULSE lines were screened for activity of the PULSE construct by the addition of exogenous d‐luciferin and exposure of plants to monochromatic red light for 4 h (Figure S3). Four selected lines confirmed for the presence and the activity of the PULSE construct were grown from gemmae in clear 24‐well plates for 15 d. After addition of exogenous d‐luciferin, the plants were placed in white light for 12 h, in red light for 12 h, and in white light for another 24 h. Images and photon counts were captured every 2 h, and photon counts were normalized by final plant mass. There was a clear increase in luminescence, and thereby of luciferase expression, following the red‐light treatment in the transgenic lines (Figure 1A). Luminescence did not increase linearly with induction time but tapered off after approximately 8 h of induction. It took approximately 6 h for luminescence to reach a steady state after the end of the induction period. The rate of decrease of luciferase activity is reflective of the half‐life of the luciferase protein rather than the off‐time of the PULSE system expression: structural changes in EL222 and PhyB are expected to result in turning off of expression on a millisecond‐to‐second scale once exposed to white light (Zoltowski et al, 2011; Heyes et al., 2019).

Luminescence signal was heterogeneously distributed in all PULSE lines, with higher levels in the apical regions of the thalli and, in some cases, with no signal detected at the center of the thallus (Figure 1A, inset). Given that the thalli of *M. polymorpha* develop from apical stem cells located in an invaginated notch at the thallus extremity (Solly et al., 2017), the apical region is expected to grow faster than the basal region of the thallus. The processes involved in growth require adenosine triphosphate (ATP) (Salin et al., 2019), and as a result the apical region of the thallus might be better able both to accumulate large amounts of protein and to provide a higher availability of ATP for the conversion of d‐luciferin to oxyluciferin.

### Induction of PULSE in response to different light conditions

The induction of expression of the PULSE system in response to different intensities of red light, different ratios of blue to red light, and different white light conditions was tested via activity of the luciferase reporter (Figure 1B–D). Three PULSE lines were grown from 36‐d‐old gemmae (red to blue light ratio) or from thallus cuttings grown for 1 wk (red light intensity and white light experiments) and were exposed to the test condition for 18 h. The PULSE system also includes *Renilla* luciferase under the control of the *A. thaliana* constitutive polyubiquitin 10 gene promoter (p*At*Ubi10) to normalize firefly luciferase readouts and thereby reduce variability due to sample handling or expression levels (Sherf et al., 1996). However, no *Renilla* luciferase was detected in any experiments carried out in this work. This could be due to inactivity of the p*At*Ubi10 promoter in *M. polymorpha*, although the promoter has been successfully used in the moss *Physcomitrium patens* (Hedw.) Mitt. (Peramuna et al., 2018). The inability to normalize luminescence data led to high levels of variability, which can likely be attributed to differences in luciferase expression between gemmae or thallus cuttings from the same line. In the absence of normalization, and to allow for comparison of data across experiments, data were expressed as micrograms of luciferase normalized by final plant mass.

The two most important metrics for an inducible system are fold‐change in expression upon induction and basal levels of expression in uninduced conditions (“leakiness”). Given that the level of expression does not increase linearly with the intensity of red light applied (Figure 1B), a red‐light intensity of 10 μmol photons·m^−2^·s^−1^ was used to determine fold‐change of expression. This value ranged from 20.6‐ to 2.9‐fold depending on the line, as determined by calculating the difference in normalized luminescence between monochromatic blue (expected to fully prevent expression) or red (expected to fully induce expression) light conditions (Figure 1C). This variability reflects leakiness through P_Opto_, where lines with lower fold‐changes in induction show basal levels of expression in monochromatic blue light.

Expression through P_Opto_ was prevented in the white light conditions used for plant growth. Plants grown in two different white light conditions, either with (W+FR) or without (W) supplemental far‐red light (see light spectra in Figure S1), had similar expression levels to conditions expected to prevent expression such as dark and monochromatic far‐red light conditions (Figure 1D).

To determine the long‐term induction dynamics of PULSE in *M. polymorpha*, two different induction strategies were tested using thallus cuttings grown for 1 wk (Figure 1E, F). When exposed to constant induction (i.e., monochromatic red light) for 6 d, activity of the luciferase reporter increased or stayed constant for a maximum of 3 d before decreasing. In two of the three lines, luciferase activity was negligible after 6 d of induction. An intermittent induction scheme was also tested; plants were placed in monochromatic red light for 3 d followed by W+FR conditions for 3 d, and this was repeated for a total of two cycles in each condition. Results show that this approach was successful at turning expression on and off, and that, apart from line #37, induction levels upon the second pulse of red light were comparable to the first.

### Phenotypic effects of the PULSE system

*Marchantia polymorpha* PULSE lines display a notable developmental phenotype characterized by slower growth, thinner and more branching thalli, increased cuticular deposition, reduced or no gemmae cup development, and lower rates of gemmae germination (Figure 1G). This phenotype is not linked to induction as it occurs in plants both before and after exposure to red light and could be seen in spores several days after germination.

Due to the lack of gemmae cup development in some lines, thallus cuttings were used to propagate plants. As can be seen in Figure 1D and 1F, this has resulted in transgene silencing in line #37, manifesting itself as a reduction in luminescence after induction, a known effect of the propagation of *M. polymorpha* by thallus cuttings.

### Using PULSE to control the biosynthesis of PHB

To demonstrate the utility of PULSE in *M. polymorpha*, we assessed its ability to regulate the expression of the heterologous PHB pathway and evaluated the effect of optogenetic induction on yield of PHB and plant health. *Marchantia polymorpha* lines constitutively expressing the three enzymes required for PHB synthesis were generated to act as a baseline against which inducible expression could be compared. A polycistronic construct for the cytosolic expression of β‐ketothiolase, acetoacetyl‐CoA reductase, and PHB synthase from *R. eutropha* was designed. The three coding sequences with stop codons removed were separated by the InteinF2A self‐cleaving fusion protein domain, composed of a Ssp DnaE mini‐intein variant engineered for hyper‐N‐terminal autocleavage that was covalently linked to the 2A peptide from the foot‐and‐mouth disease virus (Zhang et al., 2017). This construct was cloned into the p*Mp*GWB103 expression vector for constitutive expression through the endogenous p*Mp*EF1α promoter (Ishizaki et al., 2015).

Twenty‐two transgenic lines were grown for 29 d from gemmae and exhibited a large range of PHB yields and growth rates (Figure S4A, B). The most productive lines produced PHB levels similar to the highest yield of cytosolic PHB published to date, in *A. thaliana* (Poirier et al., 1995). There was a strong negative correlation (*r* = −0.61, *df* = 21, *P* = 0.0014) between average final size (measured in mm^2^) and PHB yield, supporting previous work reporting negative growth phenotypes of plants producing cytosolic PHB (Poirier et al., 1995). Thallus area is a good proxy for size in *M. polymorpha* given its predominantly two‐dimensional growth (Wang et al., 2023).

**Figure 2:**
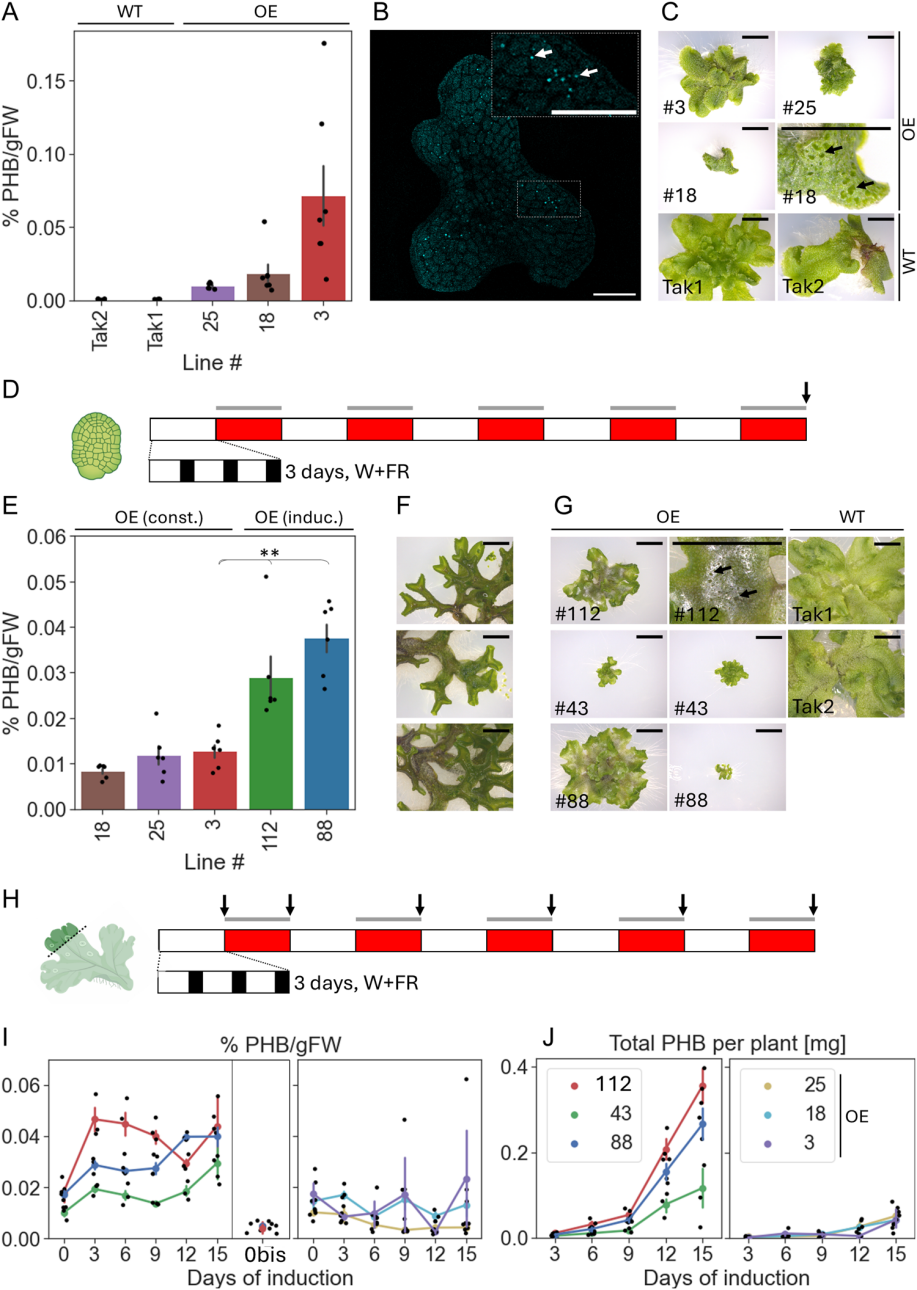
Optogenetic control over PHB production in *Marchantia polymorpha*. (A) PHB yield in three selected high‐PHB‐producing constitutive lines grown for 39 d from gemmae (*n* = 6). Error bars represent SEM. (B) Representative image of Nile Blue A–stained PHB granules in a gemma of line #25. Granules are indicated in the inset with white arrows. Scale bar is 10 μm. (C) Images of three selected high‐PHB‐producing constitutive lines and wild‐type *M. polymorpha* after 29 d of growth. Scale bar is 5 mm. Black arrows indicate enlarged pore‐like structures. (D) Induction scheme used for characterization of inducible PHB lines. The sampling point is indicated with a black arrow. (E) PHB levels expressed as percent PHB per gram fresh weight (%PHB/gFW) in both constitutive (#18, 25, 3) and inducible (#112, 88) lines grown according to the scheme shown in D (*n* = 6). Error bars represent SEM. *** represents a *P* value < 0.001, ** a *P* value < 0.01, * a *P* value < 0.05, and n.s. a *P* value > 0.05 (Mann–Whitney *U*). (F) Images of three inducible PHB lines with severe branching phenotypes that were not taken forward for further analysis. (G) Images of three selected high‐inducible PHB‐producing lines and wild‐type *M. polymorpha* after 31 d of growth and induction. Scale bar is 5 mm. Black arrows indicate enlarged pore‐like structures. (H) Induction scheme used for characterizing PHB accumulation dynamics. Sampling points are indicated with black arrows. (I) The change in PHB contents expressed in terms of %PHB/gFW of thallus cuttings from both constitutive and inducible PHB‐producing plants over time. PHB levels in inducible lines grown for the same amount of time but not induced are also shown (0bis) (*n* = 3). (J) The change in PHB contents expressed as total milligrams per plant of thallus cuttings from both constitutive and inducible PHB‐producing plants over time (*n* = 3). Legend applies to both (I) and (J), and error bars represent SEM. OE, overexpressor/transgenic lines; WT, wild‐type.

Three of the highest PHB‐yielding lines were taken forward for further analysis. Plants were grown for 39 d, and PHB yield and the expression of PHB‐producing enzymes were confirmed (Figures 2A and S4E). PHB granules were visualized in *M. polymorpha* gemmae by Nile Blue A staining (Figure 2B). As well as being smaller than the wild type (WT) (Figure S4H), all three lines had visible gaps in the dorsal epidermis not observed in WT plants (Figure 2C). Given the dependence of PHB biosynthesis on acetyl‐CoA, these structures might reflect reduced access to cytosolic acetyl‐CoA, which is essential for the acetylation of cell wall polysaccharides (Zhong et al., 2020) and the synthesis of compounds such as waxes, sterols, and cutins (Xing et al., 2014).

To generate plants with optogenetic control over PHB production, the polycistronic construct containing all three PHB‐producing enzymes separated by InteinF2A was cloned downstream of P_Opto_. Twenty‐three inducible transgenic lines were screened for their ability to produce PHB in response to 3 d in red light (Figure S4C). There was a moderate negative relationship (*r* = −0.40, *df* = 22, *P* = 0.053) between final plant size and yield of PHB (Figure S4D). Only three lines accumulated significant levels of PHB at the end of the experimental period, and the PHB accumulation levels were comparable to those in the constitutive lines (Figure 2A). Inducible expression of the PHB enzymes by monochromatic red light and minimal leakiness were demonstrated at the protein level by immunoblotting (Figure S4F). Inducible PHB lines exhibited phenotypes reminiscent of both PULSE lines and constitutive PHB lines, including branching (Figure 2F), an increase in cuticular deposition, and gaps in the dorsal epidermis (Figure 2G). These gaps were only seen in inducible lines after induction.

The three inducible lines accumulating significant levels of PHB upon induction and the previously characterized constitutive lines were grown from gemmae under the same conditions to allow for direct comparison of PHB yield. When expressed in terms of percent PHB per gram fresh weight (%PHB/gFW), the inducible lines outperformed the constitutive lines under the same conditions with 2–3‐fold higher yields (Figure 2E). Inducible control is often used in metabolic engineering to temporally separate heterologous expression and normal plant growth. We hypothesized that the negative growth phenotypes associated with PHB production might be limited by placing PHB production under inducible control. If this was the case, plants inducibly producing PHB should be larger than constitutively producing plants given the same levels of PHB accumulation. However, inducible PHB lines were roughly the same size or smaller than constitutive PHB lines grown under the same conditions (Figure S4H). For this reason, when reported as total amount of PHB per plant, there was no longer a clear advantage to the inducible strategy (Figure S4G).

One possible caveat to the comparison between inducible and constitutive PHB biosynthesis is that two different promoters were used: constitutive PHB production was driven by the p*Mp*EF1α promoter, whereas inducible PHB production was achieved by p35S‐driven expression of PULSE. However, quantification of betalain accumulation in *M. polymorpha* when expression of the biosynthesis enzymes was driven by these two promoters occurred to very similar levels (Tse et al., 2024), and hence promoter effects are unlikely to be a major factor in our experiments.

### The dynamics of PHB accumulation in constitutive and inducible PHB‐producing plants

The accumulation of PHB and plant growth over time were used to gain a fuller understanding of the interplay between growth and PHB accumulation. Due to issues generating gemmae as previously described, thallus cuttings were grown and induced according to the scheme shown in Figure 2H. For the inducible lines, some thallus cuttings were never exposed to induction conditions but were instead grown for the entire period in long‐day white light conditions, as an additional control for the proper functioning of the PULSE optogenetic system. There was very minimal PHB production in these thallus cuttings (Figure 2I), showing that the small amount of protein detected without induction (Figure S4F) was insufficient to sustain significant PHB synthesis.

The dynamics of PHB accumulation over time varied considerably between the constitutive and the inducible PHB‐producing lines. When expressed in terms of %PHB/gFW, the levels of PHB stayed constant or decreased over the experimental period in the constitutive lines but increased in the inducible lines (Figure 2I). In general, the final levels of PHB expressed as %PHB/gFW were higher in the inducible lines than in the constitutive lines, echoing results already described when plants were grown from gemmae. Whereas previous results showed no apparent benefit to inducible PHB production in terms of plant growth (Figure S4G), there was a clear increase in total milligrams of PHB per plant in the inducible lines compared to the constitutive lines when grown from thallus cuttings (Figure 2J).

### Effects of induction conditions on plant growth

The conditions used for inducing expression through PULSE were found to influence plant growth. WT and constitutive PHB lines grown with three three‐day bursts of red light were larger than plants grown only in white light (Figure S4H). The growth‐stimulating effects of red light might partly explain why the constitutive lines accumulated less PHB expressed as %PHB/gFW in induction conditions (Figure 2E) as opposed to growth in normal long‐day conditions (Figure 2A).

## DISCUSSION

### An evaluation of PULSE in *Marchantia polymorpha*

Published PULSE induction rates range from 396.5‐fold (*A. thaliana* protoplasts) to 10‐fold (stable *A. thaliana* line) (Ochoa‐Fernandez et al., 2020). In *M. polymorpha*, induction rates reached 20.6‐fold and were thus broadly similar to published results in other species. The two other important attributes of an optogenetic system are reversibility and low leakiness; reversibility of PULSE in *M. polymorpha* was demonstrated by the reduction in luciferase signal after induction when plants were placed in white light (Figure 1A, F). There was some expression through P_Opto_ in conditions expected to turn off expression (Figures 1C, S4F); however, this was insufficient to lead to significant levels of PHB accumulation (Figure 2I). As such, we can conclude that PULSE is fully functional in *M. polymorpha* and can be used to successfully drive the expression of a heterologous enzyme pathway.

Transgenic lines carrying PULSE had a prominent developmental phenotype (Figure 1G). The lack of gemmae cup formation in *M. polymorpha* PULSE lines not only reduces the ability to rapidly and easily scale up plant numbers (Ishizaki et al., 2016), it also presents significant challenges to the long‐term maintenance of PULSE lines given the propensity of thallus‐propagated plants for silencing. These phenotypic effects are present in uninduced plants and are hence likely due to the activity of one or more of the constitutively expressed PULSE construct proteins forming part of the R_on_ or B_off_ modules. Although not tested in plants on its own, the expression of an EL222 and VP16‐based optogenetic system (which forms the basis of B_off_) in zebrafish embryos resulted in minimal morphological effects or toxicity (Motta‐Mena et al., 2014).

It is therefore likely that the developmental phenotype arises due to the constitutive expression of the R_on_ module, which is derived from the *A. thaliana* PhyB and PIF6 proteins and might therefore be interfering with developmental processes in *M. polymorpha*. The *Marchantia* genome is very streamlined, with no evidence of recent whole‐genome duplications in its evolutionary history; this means that there is less genetic and regulatory complexity than in angiosperms (Bowman et al., 2017). In *Arabidopsis*, there are five different phytochromes (Phy) and eight PIFs (Cheng et al., 2021). In *M. polymorpha*, however, there is only one gene each for a phytochrome (*Mp‐Phy*) and its PIF (*Mp*‐*PIF*) (Inoue et al., 2016), and the *Mp*Phy‐*Mp*PIF pair encoded by these genes regulates a wide range of processes. For instance, *Mp*Phy and *Mp*PIF have been implicated in the germination of gemmae (Inoue et al., 2016) and in thallus and sporeling regeneration (Nishihama et al., 2015), and *Mp*PIF‐mediated signaling has been shown to regulate meristem dormancy. The meristems that develop at thallus apices are responsible for the branching architecture in *M. polymorpha*, and their relative number, position, and activity determine the final shape of the thallus (Streubel et al., 2023). The roles of *Mp*Phy and *Mp*PIF and the lack of redundancy in Phy‐PIF signaling encoded in the *M. polymorpha* genome means that the expression of another Phy‐PIF pair from *Arabidopsis* might be able to significantly interact with *Mp*Phy, *Mp*PIF, and/or their downstream targets, and that this might lead to the phenotypes observed. This is further supported by the fact that the deduced amino acid sequence of *Mp*Phy includes canonical land plant phytochrome domains and that a phylogenetic analysis of *Mp*PIF showed it to be closely related to the PIF family in *Arabidopsis* (Inoue et al., 2016).

The Phy‐PIF signaling module is evolutionarily conserved and is present in all land plants and most algal taxa (Pham et al., 2018). Although *M. polymorpha* is so far unique in possessing a single Phy‐PIF pair, the ubiquitous nature of this system in plants is likely to result in effects of PULSE on plant development in other plants too. Thus far, PULSE remains the only optogenetic system with no expression in white light that has been tested in stable plant lines. Its effects on developmental phenotype would warrant the development of an alternative system for applications in which these effects are unacceptable. Recently, another plant optogenetic system purported to be compatible with growth in white light was reported (Larsen et al., 2023). This system is specifically repressed with blue light or blue‐enriched white light, but active under other light regimes such as green‐enriched white light, and was tested in transiently transformed *N. benthamiana* leaves for the control over fluorescent protein, pigment, or immune response protein production. However, the system is considerably leaky and active in dark conditions. Despite this, and given the ability to modulate activity by changing the spectral properties of white light, this system would warrant being tested in stable plant lines.

The expression of PULSE was driven by strong constitutive p35S promoters. The library of constitutive promoters available for use in *M. polymorpha* has recently been expanded to include 367 transcription factor–derived promoters (Romani et al., 2024), some of whose relative strengths have been directly quantified for control over a heterologous biosynthetic pathway (Tse et al., 2024). Replacing the strong p35S promoters with weaker promoters might ensure adequate induction levels while minimizing developmental phenotypes. Indeed, a trade‐off might exist whereby a weaker promoter could result in higher product accumulation levels due to reduced adverse effects on plant health.

### PULSE as a tool for the metabolic engineering of *Marchantia polymorpha*

The utility of PULSE as a tool for the metabolic engineering of *M. polymorpha* was demonstrated by its successful control over PHB production, which was chosen as a case study given the established negative effects on plant growth when the PHB biosynthesis pathway is expressed constitutively (Poirier et al., 1995). It is thought that the main cause of growth reduction in plants producing cytosolic PHB is the competition between PHB and biomass‐accumulating pathways for acetyl‐CoA (Nawrath et al., 1994; Kocharin et al., 2013; McQualter et al., 2016). In metabolic engineering, inducible expression is often used in such instances to partially separate endogenous plant processes and heterologous production and thereby minimize effects on plant health. Inducibility has been used in plant‐based PHB production to avoid competition between PHB production and endogenous metabolism (Somleva et al., 2013; Mortimer, 2019; Lu et al., 2020). Chemically induced promoters such as ethanol (Lossl et al., 2005), salicylic acid (Bohmert et al., 2002), or ecdysone (Kourtz et al., 2007) have been used to drive plastidial PHB production and improve plant phenotypes. To our knowledge, however, no inducible system has been used for cytosolic PHB production. In this work, the PULSE optogenetic system was used to drive the production of cytosolic PHB in response to red light.

The production of PHB was shown to be fully inducible and minimally leaky (Figures 2I, S4F). When expressed in terms of %PHB/gFW, inducible lines consistently outperformed constitutive lines grown under the same conditions (Figure 2E, I). The unit of %PHB/gFW can be used to infer the preferential allocation of carbon and other cellular resources toward PHB given that a higher value of %PHB/gFW means a larger proportion of plant mass is composed of PHB. As such, inducible PHB production was more effective than constitutive production at re‐allocating plant resources toward PHB synthesis.

The benefit of an inducible strategy was less clear when final plant size was taken into account. When grown from thallus cuttings, PHB yield expressed as total milligrams of PHB per plant was higher in inducible lines than in constitutive lines (Figure 2J). When grown from gemmae under the same induction scheme, however, there was no longer any benefit to the inducible strategy (Figure S4G). These plants also had the same enlarged pore‐like structures after induction and showed similar levels of growth retardation to constitutive lines when compared to WT plants (Figures 2G, S4H). Expression is induced at very different developmental stages in gemmae and thallus cuttings, thus it is possible that either prolonged exposure to red light or the production of PHB affects germinating gemmae and growing thalli in different ways. These effects seem the most severe in gemmae, such that inducing the production of PHB in growing thallus tissue is clearly advantageous over both induction in the same plant lines growing from gemmae and constitutive PHB production under the same conditions.

The interpretation of benefits incurred by an inducible PHB strategy is complicated by the growth stimulation demonstrated by the induction conditions (Figure S4H). Inducing plants using three three‐day bursts of red light hence sets up an increased competition during this period between PHB and biomass production. Moreover, growth in monochromatic red light adversely affects photosynthesis (Kaiser et al., 2018); therefore, shorter pulses of red light might reduce the stimulating effect on biomass accumulation while allowing for higher PHB accumulation. Alternatively, white light supplemented with a large excess of monochromatic red light might allow for higher PHB production levels while minimizing growth stimulation.

### Concluding remarks

The PULSE system is the first published optogenetic system compatible with growth of plants in white light without promoter activation. As such, it shows promise as a tool in metabolic engineering where production often needs to be temporally restricted to minimize negative effects on the plant host, or in other fields where the need for reversibility of gene expression is crucial. In this work, the PULSE system was shown to be fully functional in the emerging metabolic engineering testbed species *M. polymorpha*, with induction fold‐changes of expression comparable to previously tested plant hosts, full reversibility, and low levels of leaky expression. The system was also modified to provide optogenetic control over the expression of a three‐enzyme heterologous pathway for PHB production, providing insights into the trade‐offs between PHB and biomass accumulation. The successful expression of both a luciferase reporter and the PHB pathway genes suggests that there is no limitation on the target genes that can be expressed using this system. Nevertheless, users should assess the reproducibility of the system using the luciferase reporter and should carry out direct quantification of their target protein, which could differ from luciferase in expression dynamics due to factors such as different turnover rates.

The wider applicability of the PULSE optogenetic system might be somewhat limited by its effect on developmental phenotype. As it is unclear whether these phenotypic effects are unique to *Marchantia* or whether they might be encountered in other plant species stably transformed with PULSE, further work is needed in a range of species to characterize these effects.

## AUTHOR CONTRIBUTIONS

A.L.L.B. and L.J.S. planned and designed the research. A.L.L.B. carried out the experimental work, analyzed the data, and wrote the first draft. A.L.L.B and L.J.S. approved the final version of the manuscript.

## Supporting information


**Figure S1.** Superimposed light spectra of conditions used for growth of *Marchantia polymorpha* in white light without (green) and with (red) supplementary far‐red light.
**Figure S2.** PULSE plasmid map.
**Figure S3.** The screening of functional *Marchantia polymorpha* transgenic PULSE lines using a photon‐counting camera.
**Figure S4.** Further characterization of the transgenic lines generated in this study.
**Table S1.** Gene parts used for genetic construct design.
**Table S2.** Primers used in this study. For a description of plasmid names, refer to Table S3.
**Table S3.** Plasmids used in this study.

## Data Availability

All data upon which conclusions made in this paper are based can be found at https://github.com/AnyaLindstromBattle/Optogenetic-control-in-Marchantia_APPS2024.

## References

[aps311632-bib-0001] Althoff, F. , S. Kopischke , O. Zobell , K. Ide , K. Ishizaki , T. Kohchi , and S. Zachgo . 2014. Comparison of the MpEF1α and CaMV35 promoters for application in *Marchantia polymorpha* overexpression studies. Transgenic Research 23(2): 235–244. 24036909 10.1007/s11248-013-9746-z

[aps311632-bib-0002] Boehm, C. R. , B. Pollak , N. Purswani , N. Patron , and J. Haseloff . 2017. Synthetic botany. Cold Spring Harbor Perspectives in Biology 9(7): a023887. 28246181 10.1101/cshperspect.a023887 PMC5495061

[aps311632-bib-0003] Bohmert, K. , I. Balbo , J. Kopka , V. Mittendorf , C. Nawrath , Y. Poirier , G. Tischendorf , et al. 2000. Transgenic *Arabidopsis* plants can accumulate polyhydroxybutyrate to up to 4% of their fresh weight. Planta 211(6): 841–845. 11144269 10.1007/s004250000350

[aps311632-bib-0004] Bohmert, K. , I. Balbo , A. Steinbuchel , G. Tischendorf , and L. Willmitzer . 2002. Constitutive expression of the β‐ketothiolase gene in transgenic plants: A major obstacle for obtaining polyhydroxybutyrate‐producing plants. Plant Physiology 128(4): 1282–1290. 11950977 10.1104/pp.010615 PMC154256

[aps311632-bib-0005] Bohmert‐Tatarev, K. , S. McAvoy , S. Daughtry , O. P. Peoples , and K. D. Snell . 2011. High levels of bioplastic are produced in fertile transplastomic tobacco plants engineered with a synthetic operon for the production of polyhydroxybutyrate. Plant Physiology 155(4): 1690–1708. 21325565 10.1104/pp.110.169581 PMC3091132

[aps311632-bib-0006] Bowman, J. L. , T. Kohchi , K. T. Yamato , J. Jenkins , S. Shu , K. Ishizaki , S. Yamaoka , et al. 2017. Insights into land plant evolution garnered from the *Marchantia polymorpha* genome. Cell 171(2): 287–304.e15. 28985561 10.1016/j.cell.2017.09.030

[aps311632-bib-0007] Cannell, N. , D. M. Emms , A. J. Hetherington , J. MacKay , S. Kelly , L. Dolan , and L. J. Sweetlove . 2020. Multiple metabolic innovations and losses are associated with major transitions in land plant evolution. Current Biology 30(10): 1783–1800. 32220326 10.1016/j.cub.2020.02.086

[aps311632-bib-0008] Cheng, M.‐C. , P. K. Kathare , I. Paik , and E. Huq . 2021. Phytochrome signaling networks. Annual Review of Plant Biology 72(1): 217–244. 10.1146/annurev-arplant-080620-024221 PMC10988782 33756095

[aps311632-bib-0009] Corrado, G. , and M. Karali . 2009. Inducible gene expression systems and plant biotechnology. Biotechnology Advances 27(6): 733–743. 19460424 10.1016/j.biotechadv.2009.05.006

[aps311632-bib-0010] Flores‐Sandoval, E. , T. Dierschke , T. J. Fisher , and J. L. Bowman . 2016. Efficient and inducible use of artificial microRNAs in *Marchantia polymorpha* . Plant and Cell Physiology 57(2): 281–290. 25971256 10.1093/pcp/pcv068

[aps311632-bib-0011] Heyes, D. J. , S. J. O. Hardman , M. N. Pedersen , J. Woodhouse , E. De La Mora , M. Wulff , M. Weik , et al. 2019. Light‐induced structural changes in a full‐length cyanobacterial phytochrome probed by time‐resolved x‐ray scattering. Communications Biology 2(1): e1. 10.1038/s42003-018-0242-0 PMC6318211 30740537

[aps311632-bib-0012] Horn, A. , A. Pascal , I. Lončarević , R. Volpatto Marques , Y. Lu , S. Miguel , F. Bourgaud , et al. 2021. Natural products from bryophytes: From basic biology to biotechnological applications. Critical Reviews in Plant Sciences 40(3): 191–217.

[aps311632-bib-0013] Hunter, J. D. 2007. Matplotlib: A 2D graphics environment. Computing in Science & Engineering 9(3): 90–95.

[aps311632-bib-0014] Inoue, K. , R. Nishihama , H. Kataoka , M. Hosaka , R. Manabe , M. Nomoto , Y. Tada , et al. 2016. Phytochrome signaling is mediated by phytochrome interacting factor in the liverwort *Marchantia polymorpha* . Plant Cell 28(6): 1406–1421. 27252292 10.1105/tpc.15.01063 PMC4944405

[aps311632-bib-0015] Ishizaki, K. , S. Chiyoda , K. T. Yamato , and T. Kohchi . 2008. *Agrobacterium*‐mediated transformation of the haploid liverwort *Marchantia polymorpha* L., an emerging model for plant biology. Plant and Cell Physiology 49(7): 1084–1091. 18535011 10.1093/pcp/pcn085

[aps311632-bib-0016] Ishizaki, K. , M. Nonomura , H. Kato , K. T. Yamato , and T. Kohchi . 2012. Visualization of auxin‐mediated transcriptional activation using a common auxin‐responsive reporter system in the liverwort *Marchantia polymorpha* . Journal of Plant Research 125(5): 643–651. 22311005 10.1007/s10265-012-0477-7

[aps311632-bib-0017] Ishizaki, K. , R. Nishihama , M. Ueda , K. Inoue , S. Ishida , Y. Nishimura , T. Shikanai , and T. Kohchi . 2015. Development of Gateway binary vector series with four different selection markers for the liverwort *Marchantia polymorpha* . PLoS ONE 10(9): e0138876. 26406247 10.1371/journal.pone.0138876 PMC4583185

[aps311632-bib-0018] Ishizaki, K. , R. Nishihama , K. T. Yamato , and T. Kohchi . 2016. Molecular genetic tools and techniques for *Marchantia polymorpha* research. Plant and Cell Physiology 57(2): 262–270. 26116421 10.1093/pcp/pcv097

[aps311632-bib-0019] Kaiser, E. , T. Ouzounis , H. Giday , R. Schipper , E. Heuvelink , and L. F. M. Marcelis . 2018. Adding blue to red supplemental light increases biomass and yield of greenhouse‐grown tomatoes, but only to an optimum. Frontiers in Plant Science 9: e2002. 10.3389/fpls.2018.02002 PMC6339924 30693012

[aps311632-bib-0020] Karimi, M. , D. Inzé , and A. Depicker . 2002. GATEWAY™ vectors for *Agrobacterium*‐mediated plant transformation. Trends in Plant Science 7(5): 193–195. 11992820 10.1016/s1360-1385(02)02251-3

[aps311632-bib-0021] Kluyver, T. , B. Ragan‐Kelley , F. Pérez , B. Granger , M. Bussonnier , J. Frederic , K. Kelley , et al. 2016. Jupyter Notebooks—a publishing format for reproducible computational workflows. *In* F. Loizides and B. Schmidt [eds.], Positioning and power in academic publishing: Players, agents and agendas, 87–90. IOS Press, Amsterdam, the Netherlands.

[aps311632-bib-0022] Kocharin, K. , V. Siewers , and J. Nielsen . 2013. Improved polyhydroxybutyrate production by *Saccharomyces cerevisiae* through the use of the phosphoketolase pathway. Biotechnology and Bioengineering 110(8): 2216–2224. 23456608 10.1002/bit.24888

[aps311632-bib-0023] Koncz, C. , and J. Schell . 1986. The promoter of TL‐DNA gene 5 controls the tissue‐specific expression of chimaeric genes carried by a novel type of *Agrobacterium* binary vector. Molecular and General Genetics 204(3): 383–396.

[aps311632-bib-0024] Kourtz, L. , K. Dillon , S. Daughtry , O. P. Peoples , and K. D. Snell . 2007. Chemically inducible expression of the PHB biosynthetic pathway in *Arabidopsis* . Transgenic Research 16(6): 759–769. 17279436 10.1007/s11248-007-9067-1

[aps311632-bib-0025] Kubota, A. , K. Ishizaki , M. Hosaka , and T. Kohchi . 2013. Efficient *Agrobacterium*‐mediated transformation of the liverwort *Marchantia polymorpha* using regenerating thalli. Bioscience Biotechnology and Biochemistry 77(1): 167–172. 23291762 10.1271/bbb.120700

[aps311632-bib-0026] Larsen, B. , R. Hofmann , I. S. Camacho , R. W. Clarke , J. C. Lagarias , A. R. Jones , and A. M. Jones . 2023. Highlighter: An optogenetic system for high‐resolution gene expression control in plants. PLoS Biology 21(9): e3002303. 37733664 10.1371/journal.pbio.3002303 PMC10513317

[aps311632-bib-0027] Lossl, A. , K. Bohmert , H. Harloff , C. Eibl , S. Muhlbauer , and H. U. Koop . 2005. Inducible trans‐activation of plastid transgenes: Expression of the *R. eutropha phb* operon in transplastomic tobacco. Plant and Cell Physiology 46(9): 1462–1471. 15964903 10.1093/pcp/pci157

[aps311632-bib-0028] Lu, H. , G. Yuan , S. H. Strauss , T. J. Tschaplinski , G. A. Tuskan , J.‐G. Chen , and X. Yang . 2020. Reconfiguring plant metabolism for biodegradable plastic production. BioDesign Research 2020: e9078303. 10.34133/2020/9078303 PMC10530661 37849903

[aps311632-bib-0029] Mann, H. B. , and D. R. Whitney . 1947. On a test of whether one of two random variables is stochastically larger than the other. Annals of Mathematical Statistics 18: 50–60.

[aps311632-bib-0030] McQualter, R. B. , L. A. Petrasovits , L. K. Gebbie , D. Schweitzer , D. M. Blackman , P. Chrysanthopoulos , M. P. Hodson , et al. 2015. The use of an acetoacetyl‐CoA synthase in place of a β‐ketothiolase enhances poly‐3‐hydroxybutyrate production in sugarcane mesophyll cells. Plant Biotechnology Journal 13(5): 700–707. 25532451 10.1111/pbi.12298

[aps311632-bib-0031] McQualter, R. B. , C. Bellasio , L. K. Gebbie , L. A. Petrasovits , R. W. Palfreyman , M. P. Hodson , M. R. Plan , et al. 2016. Systems biology and metabolic modelling unveils limitations to polyhydroxybutyrate accumulation in sugarcane leaves; lessons for C_4_ engineering. Plant Biotechnology Journal 14(2): 567–580. 26015295 10.1111/pbi.12399 PMC11629826

[aps311632-bib-0032] Mortimer, J. C. 2019. Plant synthetic biology could drive a revolution in biofuels and medicine. Experimental Biology and Medicine 244(4): 323–331. 30249124 10.1177/1535370218793890 PMC6435885

[aps311632-bib-0033] Motta‐Mena, L. B. , A. Reade , M. J. Mallory , S. Glantz , O. D. Weiner , K. W. Lynch , and K. H. Gardner . 2014. An optogenetic gene expression system with rapid activation and deactivation kinetics. Nature Chemical Biology 10(3): 196–202. 24413462 10.1038/nchembio.1430 PMC3944926

[aps311632-bib-0034] Nawrath, C. , Y. Poirier , and C. Somerville . 1994. Targeting of the polyhydroxybutyrate biosynthetic‐pathway to the plastids of *Arabidopsis thaliana* results in high‐levels of polymer accumulation. Proceedings of the National Academy of Sciences, USA 91(26): 12760–12764. 10.1073/pnas.91.26.12760 PMC45519 7809117

[aps311632-bib-0035] Nishihama, R. , K. Ishizaki , M. Hosaka , Y. Matsuda , A. Kubota , and T. Kohchi . 2015. Phytochrome‐mediated regulation of cell division and growth during regeneration and sporeling development in the liverwort *Marchantia polymorpha* . Journal of Plant Research 128(3): 407–421. 25841334 10.1007/s10265-015-0724-9

[aps311632-bib-0036] Nishihama, R. , S. Ishida , H. Urawa , Y. Kamei , and T. Kohchi . 2016. Conditional gene expression/deletion systems for *Marchantia polymorpha* using its own heat‐shock promoter and Cre/*lox* P‐mediated site‐specific recombination. Plant and Cell Physiology 57(2): 271–280. 26148498 10.1093/pcp/pcv102

[aps311632-bib-0037] Ochoa‐Fernandez, R. , N. B. Abel , F. G. Wieland , J. Schlegel , L. A. Koch , J. B. Miller , R. Engesser , et al. 2020. Optogenetic control of gene expression in plants in the presence of ambient white light. Nature Methods 17(7): 717–725. 32601426 10.1038/s41592-020-0868-y

[aps311632-bib-0038] Oliver, D. J. , B. J. Nikolau , and E. S. Wurtele . 2009. Acetyl‐CoA: Life at the metabolic nexus. Plant Science 176(5): 597–601.

[aps311632-bib-0039] Peramuna, A. , H. Bae , E. K. Rasmussen , B. Dueholm , T. Waibel , J. H. Critchley , K. Brzezek , et al. 2018. Evaluation of synthetic promoters in *Physcomitrella patens* . Biochemical and Biophysical Research Communications 500(2): 418–422. 29660341 10.1016/j.bbrc.2018.04.092

[aps311632-bib-0040] Pham, V. N. , P. K. Kathare , and E. Huq . 2018. Phytochromes and phytochrome interacting factors. Plant Physiology 176(2): 1025–1038. 29138351 10.1104/pp.17.01384 PMC5813575

[aps311632-bib-0041] Poirier, Y. , D. E. Dennis , K. Klomparens , and C. Somerville . 1992. Polyhydroxybutyrate, a biodegradable thermoplastic, produced in transgenic plants. Science 256(5056): 520–523. 17787950 10.1126/science.256.5056.520

[aps311632-bib-0042] Poirier, Y. , C. Somerville , L. A. Schechtman , M. M. Satkowski , and I. Noda . 1995. Synthesis of high‐molecular‐weight poly([r]‐(‐)‐3‐hydroxybutyrate) in transgenic *Arabidopsis thaliana* plant‐cells. International Journal of Biological Macromolecules 17(1): 7–12. 7772565 10.1016/0141-8130(95)93511-u

[aps311632-bib-0043] Porebski, S. , L. G. Bailey , and B. R. Baum . 1997. Modification of a CTAB DNA extraction protocol for plants containing high polysaccharide and polyphenol components. Plant Molecular Biology Reporter 15(1): 8–15.

[aps311632-bib-0044] Puigbo, P. , E. Guzman , A. Romeu , and S. Garcia‐Vallve . 2007. OPTIMIZER: A web server for optimizing the codon usage of DNA sequences. Nucleic Acids Research 35: W126–W131. 17439967 10.1093/nar/gkm219 PMC1933141

[aps311632-bib-0045] Romani, F. , S. Sauret‐Güeto , M. Rebmann , D. Annese , I. Bonter , M. Tomaselli , T. Dierschke , et al. 2024. The landscape of transcription factor promoter activity during vegetative development in *Marchantia* . The Plant Cell 36(6): 2140–2159. 38391349 10.1093/plcell/koae053 PMC11132968

[aps311632-bib-0046] Salin, K. , E. M. Villasevil , G. J. Anderson , S. G. Lamarre , C. A. Melanson , I. McCarthy , C. Selman , and N. B. Metcalfe . 2019. Differences in mitochondrial efficiency explain individual variation in growth performance. Proceedings of the Royal Society B: Biological Sciences 286(1909): 20191466. 10.1098/rspb.2019.1466 PMC6732382 31431161

[aps311632-bib-0047] Sauret‐Gueto, S. , E. Frangedakis , L. Silvestri , M. Rebmann , M. Tomaselli , K. Markel , M. Delmans , et al. 2020. Systematic tools for reprogramming plant gene expression in a simple model, *Marchantia polymorpha* . ACS Synthetic Biology 9(4): 864–882. 32163700 10.1021/acssynbio.9b00511

[aps311632-bib-0048] Sherf, B. A. , S. L. Navarro , R. R. Hannah , and K. V. Wood . 1996. Dual‐luciferase^TM^ reporter assay: An advanced co‐reporter technology integrating firefly and *Renilla* luciferase assays. Promega Notes Magazine 57: 2.

[aps311632-bib-0049] Shikata, H. , and P. Denninger . 2022. Plant optogenetics: Applications and perspectives. Current Opinion in Plant Biology 68: e102256. 10.1016/j.pbi.2022.102256 35780691

[aps311632-bib-0050] Solly, J. E. , N. J. Cunniffe , and C. J. Harrison . 2017. Regional growth rate differences specified by apical notch activities regulate liverwort thallus shape. Current Biology 27(1): 16–26. 27939317 10.1016/j.cub.2016.10.056 PMC5226888

[aps311632-bib-0051] Somleva, M. N. , O. P. Peoples , and K. D. Snell . 2013. PHA bioplastics, biochemicals, and energy from crops. Plant Biotechnology Journal 11(2): 233–252. 23294864 10.1111/pbi.12039

[aps311632-bib-0052] Streubel, S. , S. Deiber , J. Rötzer , M. Mosiolek , K. Jandrasits , and L. Dolan . 2023. Meristem dormancy in *Marchantia polymorpha* is regulated by a liverwort‐specific miRNA and a clade III *SPL* gene. Current Biology 33(4): 660–674.e4. 36696899 10.1016/j.cub.2022.12.062

[aps311632-bib-0053] Sugano, S. S. , M. Shirakawa , J. Takagi , Y. Matsuda , T. Shimada , I. Hara‐Nishimura , and T. Kohchi . 2014. CRISPR/Cas9‐mediated targeted mutagenesis in the liverwort *Marchantia polymorpha* L. Plant and Cell Physiology 55(3): 475–481. 24443494 10.1093/pcp/pcu014

[aps311632-bib-0054] Tse, S. W. , D. Annese , F. Romani , F. Guzman‐Chavez , I. Bonter , E. Forestier , E. Frangedakis , and J. Haseloff . 2024. Optimising promoters and subcellular localisation for constitutive transgene expression in *Marchantia polymorpha* . Plant and Cell Physiology 65(8): 1298–1309. 38822700 10.1093/pcp/pcae063 PMC11369823

[aps311632-bib-0055] Tsuboyama, S. , and Y. Kodama . 2018. AgarTrap protocols on your benchtop: Simple methods for *Agrobacterium*‐mediated genetic transformation of the liverwort *Marchantia polymorpha* . Plant Biotechnology 35(2): 93–99. 31819711 10.5511/plantbiotechnology.18.0312b PMC6879393

[aps311632-bib-0056] Tsuboyama‐Tanaka, S. , and Y. Kodama . 2015. AgarTrap‐mediated genetic transformation using intact gemmae/gemmalings of the liverwort *Marchantia polymorpha* L. Journal of Plant Research 128(2): 337–344. 25663453 10.1007/s10265-014-0695-2

[aps311632-bib-0057] Virtanen, P. , R. Gommers , T. E. Oliphant , M. Haberland , T. Reddy , D. Cournapeau , E. Burovski , et al. 2020. SciPy 1.0: Fundamental algorithms for scientific computing in Python. Nature Methods 17(3): 261–272. 32015543 10.1038/s41592-019-0686-2 PMC7056644

[aps311632-bib-0058] Wang, L. , M.‐C. Wan , R.‐Y. Liao , J. Xu , Z.‐G. Xu , H.‐C. Xue , Y.‐X. Mai , and J.‐W. Wang . 2023. The maturation and aging trajectory of *Marchantia polymorpha* at single‐cell resolution. Developmental Cell 58(15): 1429–1444.e6. 37321217 10.1016/j.devcel.2023.05.014

[aps311632-bib-0059] Waskom, M. 2021. seaborn: Statistical data visualization. Journal of Open Source Software 6(60): 3021.

[aps311632-bib-0060] Xing, S. F. , N. van Deenen , P. Magliano , L. Frahm , E. Forestier , C. Nawrath , H. Schaller , et al. 2014. ATP citrate lyase activity is post‐translationally regulated by sink strength and impacts the wax, cutin and rubber biosynthetic pathways. The Plant Journal 79(2): 270–284. 24844815 10.1111/tpj.12559

[aps311632-bib-0061] Zhang, B. , M. Rapolu , S. Kumar , M. Gupta , Z. B. Liang , Z. L. Han , P. Williams , and W. W. Su . 2017. Coordinated protein co‐expression in plants by harnessing the synergy between an intein and a viral 2A peptide. Plant Biotechnology Journal 15(6): 718–728. 27879048 10.1111/pbi.12670 PMC5425387

[aps311632-bib-0062] Zhong, R. , D. Cui , E. A. Richardson , D. R. Phillips , P. Azadi , G. Lu , and Z.‐H. Ye . 2020. Cytosolic acetyl‐CoA generated by ATP‐citrate lyase is essential for acetylation of cell wall polysaccharides. Plant and Cell Physiology 61(1): 64–75. 31503286 10.1093/pcp/pcz178

[aps311632-bib-0063] Zoltowski, B. D. , A. I. Nash , and K. H. Gardner . 2011. Variations in protein–flavin hydrogen bonding in a light, oxygen, voltage domain produce non‐Arrhenius kinetics of adduct decay. Biochemistry 50(41): 8771–8779. 21923139 10.1021/bi200976a PMC3381950

