# Supplementary material for: Optogenetic control of transgene expression in Marchantia polymorpha
Source: Appl Plant Sci. 2025 Jan 28;13(4):e11632. doi: 10.1002/aps3.11632 (PMC12319710; doi:10.1002/aps3.11632)
Supplement: Supplementary file 1 — Figure S1. Superimposed light spectra of conditions used for growth of Marchantia polymorpha in white light without (green) and with (red) supplementary far‐red light. Figure S2. PULSE plasmid map. Figure S3. The screening of functional Marchantia polymorpha transgenic PULSE lines using a photon‐counting camera. Figure S4. Further characterization of the transgenic lines generated in this study. Table S1. Gene parts used for genetic construct design. Table S2. Primers used in this study. For a description of plasmid names, refer to Table S3. Table S3. Plasmids used in this study. [file APS3-13-e11632-s001.docx]

**Supporting information for “Optogenetic control of transgene expression in *Marchantia polymorpha*”**

**
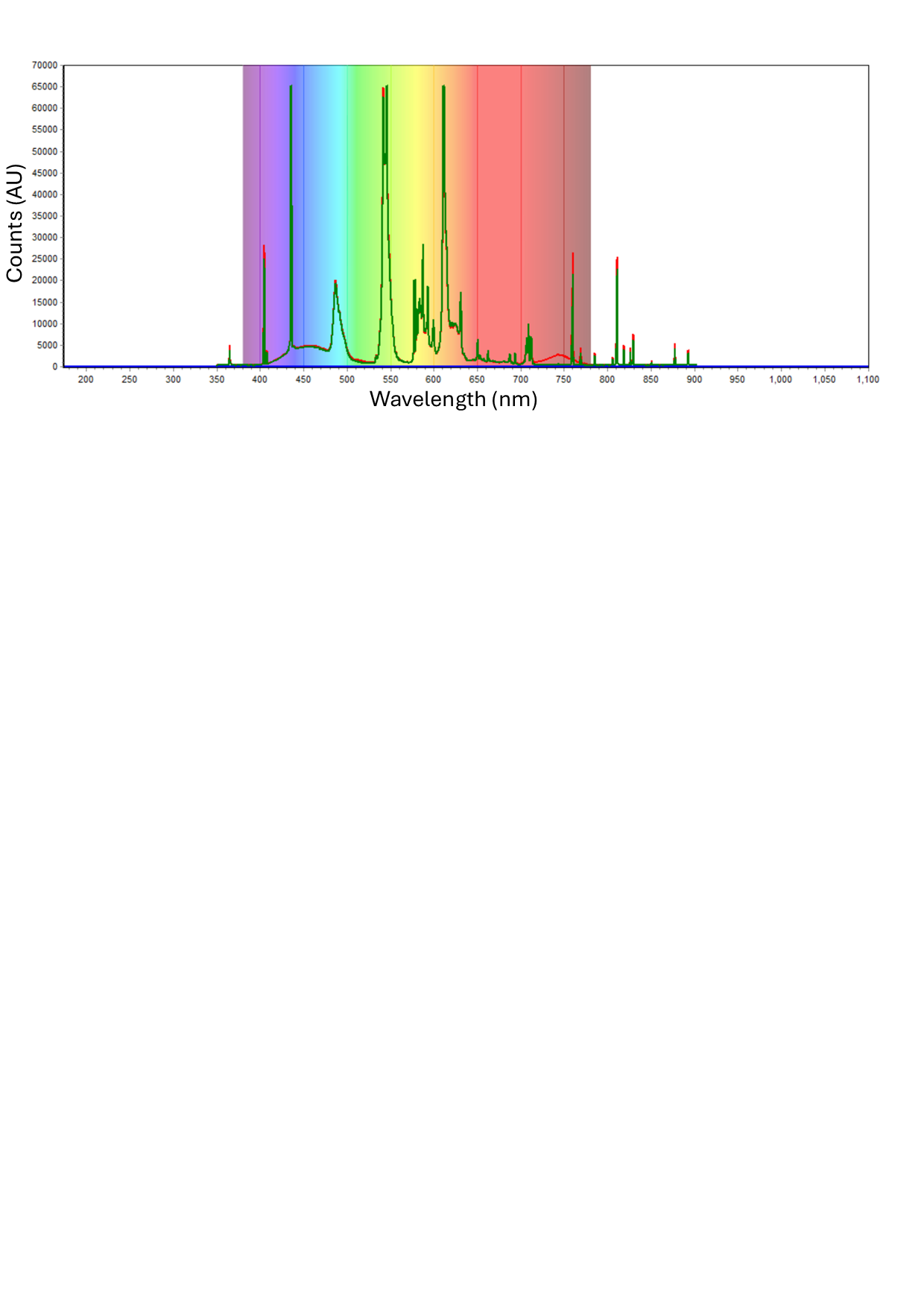
**

**Figure S1.** Superimposed light spectra of conditions used for growth of *Marchantia polymorpha* in white light without (green) and with (red) supplementary far-red light.

**Table S1.** Gene parts used for genetic construct design.

| **Name** | **Description** | **Sequence source or protein sequence** |
| --- | --- | --- |
| PULSE | PULSE is an optogenetic system that is inactive under white light and activated with monochromatic red light (Ochoa-Fernandez et al., 2020). Carries resistance to kanamycin. For more information, see section “Adaptation of the PULSE optogenetic system for use in *Marchantia polymorpha*.” | Part ID JPUB_016925 from the Joint BioEnergy Institute ICE repository |
| HygR | Hygromycin resistance cassette | Addgene plasmid number 71536 |
| PhbA | Coding sequence for β-ketothiolase from *Ralstonia eutropha* | GenBank accession number FJ897461.1 |
| PhbB | Coding sequence for acetoacetyl-CoA reductase from *R. eutropha* | GenBank accession number FJ897462.1 |
| PhbC | Coding sequence for PHB synthase from *R. eutropha* | GenBank accession number MH558939.1 |
| InteinF2A | Self-cleaving fusion protein domain composed of a *Ssp* DnaE mini-intein variant engineered for hyper-N-terminal autocleavage which is covalently linked to the F2A peptide (self-cleaving 2A peptide from foot-and-mouth disease virus) (Zhang et al., 2017). | (Zhang et al., 2017) |
| GSG linker | A glycine-serine-glycine (GSG) linker fused to the N-terminus of 2A peptides, which can improve cleavage efficiency. | (Wang et al., 2015) |
| FLAG | Short hydrophilic protein tag used to detect proteins in western blotting | DYKDDDDK |
| His | Polyhistidine tag enabling purification of the constructs using Ni-affinity purification. | HHHHHHHH or HHHHHHHHHH |
| Myc | Short protein tag derived from the *c-myc* gene product and used to detect proteins in western blotting | EQKLISEEDL |
| HA | Short protein tag derived from the human influenza hemagglutinin surface glycoprotein and used to detect proteins in western blotting. | YPYDVPDYA |

**REFERENCES**

Ochoa-Fernandez, R., N. B. Abel, F. G. Wieland, J. Schlegel, L. A. Koch, J. B. Miller, R. Engesser, et al. 2020. Optogenetic control of gene expression in plants in the presence of ambient white light. *Nature Methods* 17(7): 717–725.

Wang, Y. C., F. Wang, R. Y. Wang, P. Zhao, and Q. Y. Xia. 2015. 2A self-cleaving peptide-based multi-gene expression system in the silkworm *Bombyx mori*. *Scientific Reports* 5: e16273.

Zhang, B., M. Rapolu, S. Kumar, M. Gupta, Z. B. Liang, Z. L. Han, P. Williams, and W. W. Su. 2017. Coordinated protein co-expression in plants by harnessing the synergy between an intein and a viral 2A peptide. *Plant Biotechnology Journal* 15(6): 718–728.

**Table S2.** Primers used in this study. For a description of plasmid names, refer to Table S3.

| **Name** | **Primer sequence** | **Description** |
| --- | --- | --- |
| Sequencing and genotyping | | |
| M13F | GTAAAACGACGGCCAGT | In-house sequencing primer M13F, binding in cloning vector backbone |
| M13R | CAGGAAACAGCTATGAC | In-house sequencing primer M13R, binding in cloning vector backbone |
| AS_seq_09F_alt | GGAAGTTCATTTCATTTGGAGA | Sequencing primer for pK7WG2 vector binding in p35S. Courtesy of András Sándor. |
| AS_seq_011R | AGGTCACTGGATTTTGGTTTTAG | Sequencing primer for pK7WG2 vector binding in T35S. Courtesy of András Sándor. |
| ALB_seq_FLuc | CAGCCTAATGACTAGTATGGAA | Sequencing primer for PULSE and PULSE_HygR binding in FLuc |
| ALB_seq_PhyB | GAGAACAAGCTCAATCGTCG | Sequencing primer for PULSE and PULSE_HygR binding in PhyB |
| ALB_seq_RLuc | TGTCGCGAATGGCTTCGAAAGT | Sequencing primer for PULSE and PULSE_HygR binding in RLuc |
| ALB_seq_4Frag3_HygR | ATTCGGCGTTAATTCAGTAC | Sequencing primer for PULSE_HygR binding in HygR |
| ALB_seq_HygR_4Frag1 | CAAATTGACGCTTAGACAAC | Sequencing primer for PULSE and PULSE_HygR binding upstream of left border |
| ALB_seq_4Frag1_4Frag2 | TCTTCCAGCGGATAGAATGG | Sequencing primer for PULSE and PULSE_HygR binding in FLuc |
| ALB_seq_4Frag2_4Frag3 | CGATTTCGATCTGGACATG | Sequencing primer for PULSE and PULSE_HygR binding in VP16 |
| ALB_seq_PhyBdel_fwd | GTGGTTACATTCAGCCTTTCG | Sequencing primer for PULSE and PULSE_HygR binding in PhyB |
| ALB_seq_PhyBdel_rev | CTGTAAACCGAAAGCCTGC | Sequencing primer for PULSE and PULSE_HygR binding in PhyB |
| oROF422 | AGAACTGCCTGCGTGAGATT | Genotyping primer for PULSE_HygR binding in FLuc |
| oROF423 | TTTTCCGTCATCGTCTTTCC | Genotyping primer for PULSE_HygR binding in FLuc |
| ALB_seq_PhbA_PhbB | TCTGGTGACCTTGCTGCATG | Sequencing primer for ConstPHB_InteinF2A, genotyping primer for ConstPHB_InteinF2A and PULSE_HygR_Phb; binds in PhbA |
| ALB_IntPhbgeno_rev | ATGAAATCGAAACCAAGTGCC | Genotyping primer for ConstPHB_InteinF2A and PULSE_HygR_Phb binding in PhbB |
| ALB_seq_PhbC_PhbB | CTGGGGTCACTTTGAAAGGC | Sequencing primer for ConstPHB_InteinF2A binding in PhbC |
| ALB_seq_PhbCnonsense_fwd | GACGGAGGCTCGACATAAG | Sequencing primer for ConstPHB_InteinF2A binding in InteinF2A |
| ALB_seq_PhbCnonsense_rev | GAGTGAACTCTCAGGTTG | Sequencing primer for ConstPHB_InteinF2A binding in PhbC |
| ALB_seq_M13Ralt | GTTAGCTCACTCATTAGGCAC | Alternative M13R sequencing primer binding in backbone of p*Mp*GWB Gateway vectors |
| ALB_seq_Frag3I_Frag4 | ACCTACATTTACGGGAGCAG | Sequencing primer for PULSE_HygR_Phb binding in PhbC |
| Gibson assembly | | |
| HygR_fwd | AATTCTTGCGGAGGAGCTAACTCACATTAGGC | Primers used for the assembly of PULSE_HygR from p*Mp*GE010_g1_BHLH51 and PULSE |
| HygR_rev | CGTCGATCTAGTTTGCGTATTGGCTAGAGC |  |
| JPUB4frag_1_fwd | AGCCAATACGCAAACTAGATCGACGCTGGATC |  |
| JPUB4frag_1_rev | GCGATCTGACGGTTCACTAAACGAGCTCTGC |  |
| JPUB4frag_2_fwd | CTCGTTTAGTGAACCGTCAGATCGCCTGGAG |  |
| JPUB4frag_2_rev | ATCGGTAAACATCTGCTCAAACTCGAAGTCGG |  |
| JPUB4frag_3_fwd | CGAGTTTGAGCAGATGTTTACCGATGCCCTTG |  |
| JPUB4frag_3_rev | GTGAGTTAGCTCCTCCGCAAGAATTCAAGC |  |
| Backbone_rev | ACGACAAGAGGGCACTTCCTTATTCCAG |  |
| ConstrInt1_fwd | ATAAGGAAGTGCCCTCTTGTCGTCGGCCAAATAATG | Primers used for the assembly of ConstPHB_InteinF2A from genes synthesized by TWIST |
| Constr1Int_rev | GGCAAGCCTGCTTAAGATCCTCCTCGGATATAAGTTTC |  |
| Constr3Int_fwd | GAGGAGGATCTTAAGCAGGCTTGCCTTTCTTTC |  |
| Constr3Int_rev | GTGGTGGTGATGGTGATGATGGTGGTGATGC |  |
| Constr2Int_fwd | CCACCATCATCACCATCACCACCACATGGCC |  |
| Constr2Int_rev | ACCATCGCAGACTTATCATCACTGAGCCTCCAC |  |
| BackboneInt_fwd | TCAGTGATGATAAGTCTGCGATGGTGGATG |  |
| BackboneInt_rev | CGACGACAAGAGGGCACTTCCTTATTCCAG |  |
| AtALB_rev | TCCGAAATGAGCTTCTGCTCCTTGGTGTACAACACATC |  |
| BB_AttDt_fwd | GAGCAGAAGCTCATTTCG |  |
| BB_AttDt_rev | ATTTCAAAACATAGCCTGCTTTTTTGTAC |  |
| PULSE_HygR_frag1_fwd | AATTCGTAAGGAGGTTACTAGATCGACGCTGAG | Primers used for the assembly of PULSE_HygR_Phb from PULSE_HygR and ConstPHB_InteinF2A |
| PULSE_HygR_frag1_rev | ATTGTGGTGTAAACAAATTGACGCTTAGACAAC |  |
| PULSE_HygR_frag2_fwd | AGCGTCAATTTGTTTACACCACAATATATCCTGCC |  |
| PULSE_HygR_frag2_rev | ATGCATAGCCTGACTAGTCATTAGGCTGGATC |  |
| InteinF2A_allPHB_fwd | CCTAATGACTAGTCAGGCTATGCATCATCACC |  |
| InteinF2A_allPHB_rev | GAAGCCCTGCAGGTTAAGCGTAGTCCGGAAC |  |
| PULSE_HygR_frag4_fwd | GACTACGCTTAACCTGCAGGGCTTCTCTAG |  |
| PULSE_HygR_frag4_rev | CGATCTAGTAACCTCCTTACGAATTCCCATGG |  |

**Table S3.** Plasmids used in this study.

| **Name** | **Description** | **Source** |
| --- | --- | --- |
| pK7WG2 | Gateway expression vector for overexpression in plants using the Cauliflower Mosaic Virus 35S promoter (p35S). Carries a kanamycin resistance gene. | Gift from Francesco Licausi |
| p*Mp*GWB103 | Gateway expression vector for overexpression in *Marchantia polymorpha* using the p*Mp*EF1α promoter. Carries a hygromycin resistance gene. | Gift from Takayuki Kohchi |
| p*Mp*GE010_g1_BHLH51 | Source of hygromycin resistance cassette used for cloning of PULSE_HygR | Gift from Francesco Licausi |
| PULSE | PULSE optogenetic system, carries kanamycin resistance gene. | Gift from Matias Zurbriggen |
| PULSE_HygR | As PULSE but with a hygromycin resistance gene. | This study |
| PULSE_HygR_Phb | As PULSE_HygR but firefly luciferase coding sequence replaced with the cassette coding for PhbA, PhbB, and PhbC separated by the InteinF2A viral self-cleaving peptide. | This study |
| ConstPHB_InteinF2A | Cloning vector containing the cassette coding for PhbA, PhbB, and PhbC separated by the InteinF2A viral self-cleaving peptide | This study |
| GWB103_ConstPHB_InteinF2A | As ConstPHB_InteinF2A but in the p*Mp*GWB103 expression vector | This study |

PhbA: β-ketothiolase, PhbB: acetoacetyl-CoA reductase, PhbC: PHB synthase.


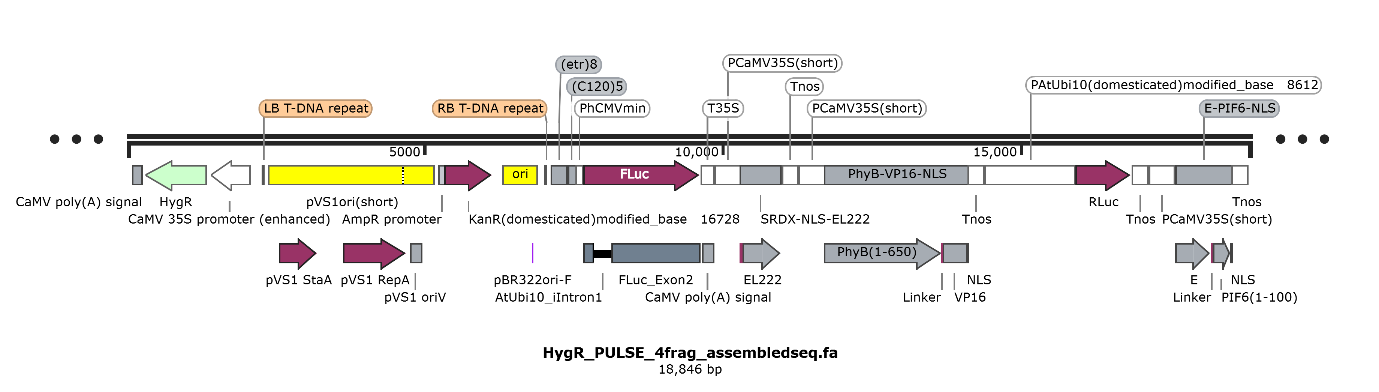
**Figure S2.** PULSE plasmid map.


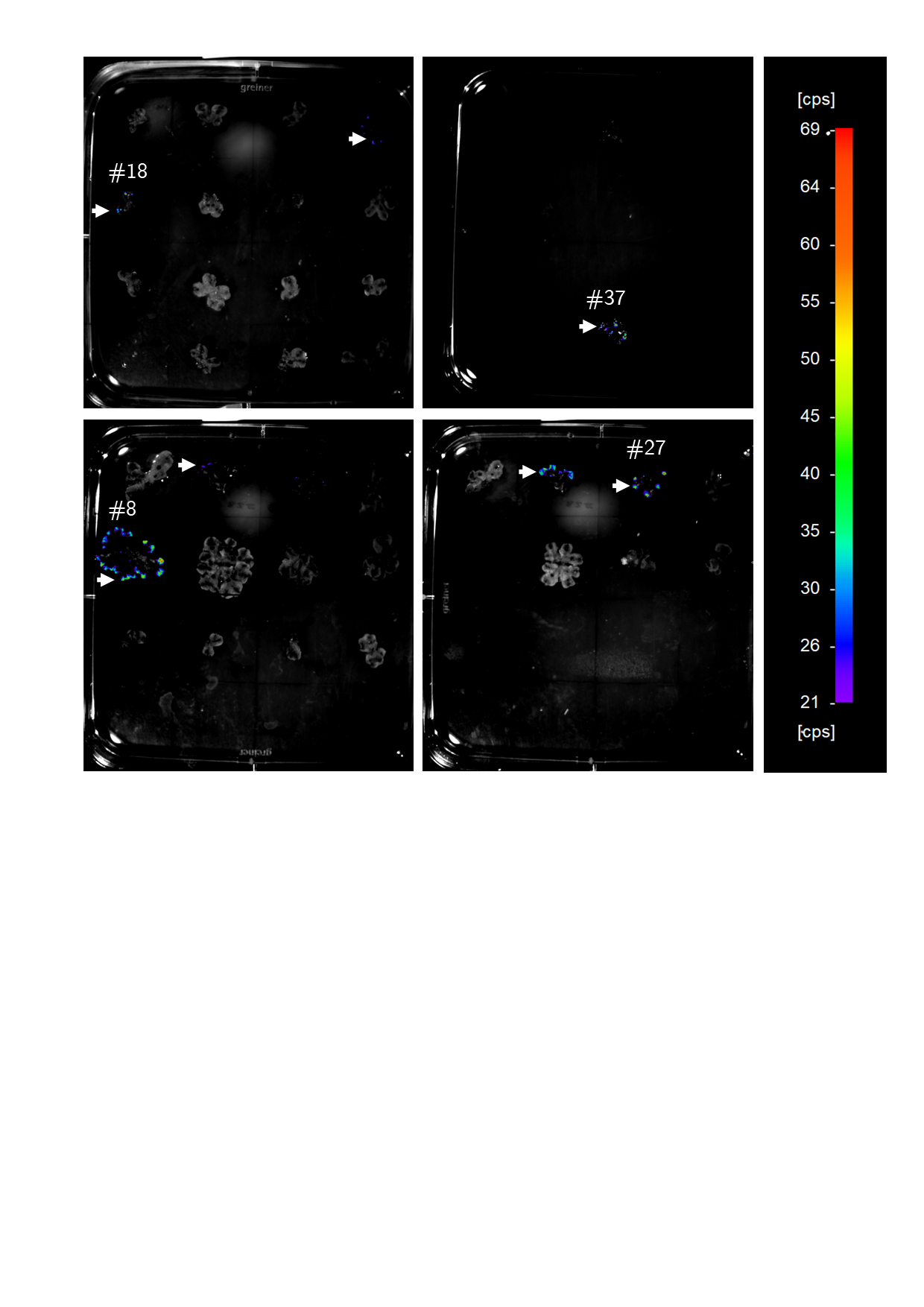


**Figure S3.** The screening of functional *Marchantia polymorpha* transgenic PULSE lines using a photon-counting camera. Luminescence is indicated by white arrows, with the lines chosen for downstream analysis specified.


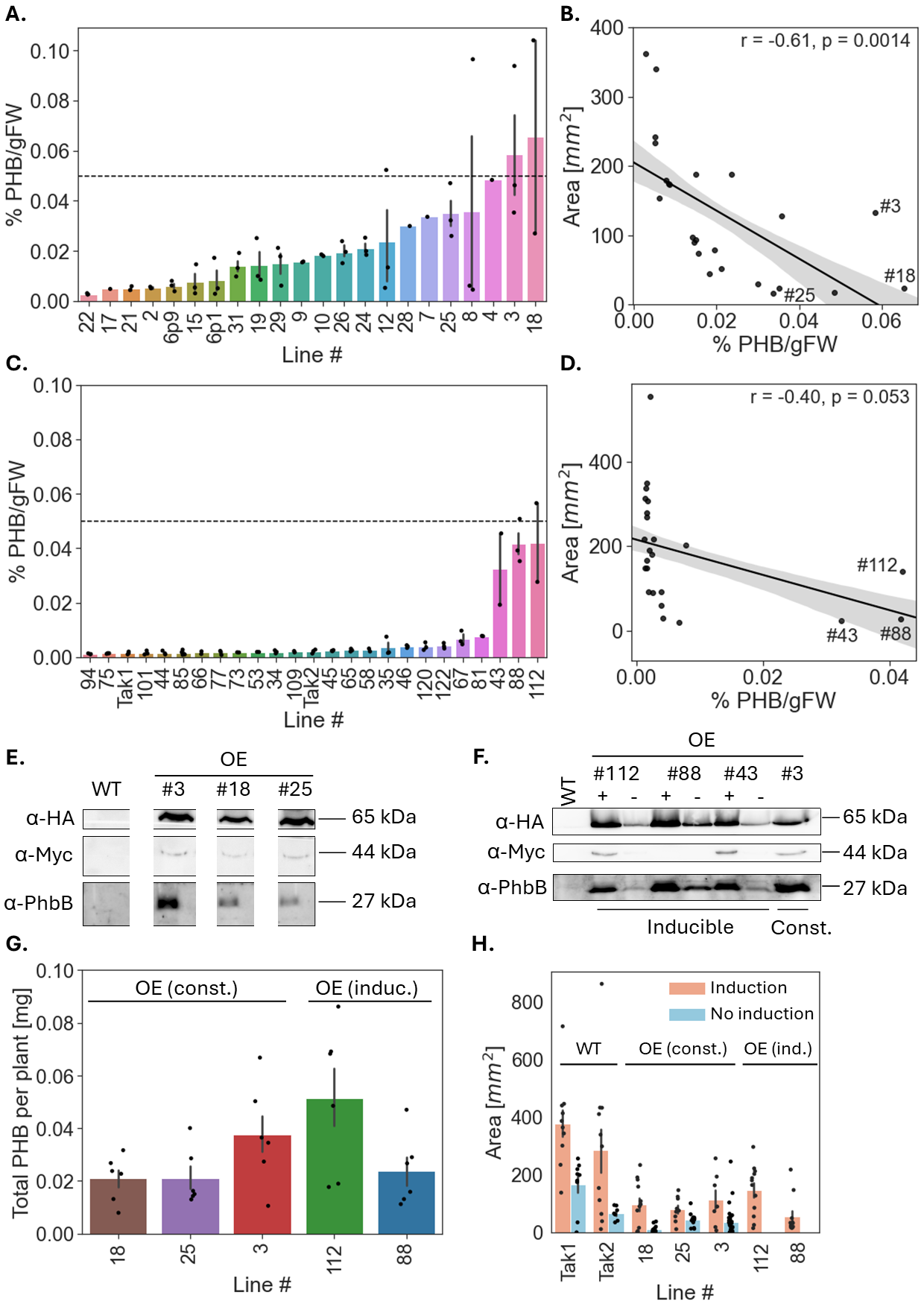


**Figure S4.** Further characterization of the transgenic lines generated in this study. (A) Twenty-two constitutive PHB lines were screened for PHB production in terms of percent PHB per gram fresh weight (%PHB/gFW). Dotted line represents the highest published cytosolic PHB yield in a plant (*n* = 1–3). Error bars indicate standard error of the mean (SEM). (B) The relationship between PHB production and final plant size in mm^2^ after 29-d growth in the 22 screened lines. Average values for each line are plotted. Shaded area represents SEM. Pearson correlation coefficient (*r*) and *P* value (p) shown in top-right corner. (C) Twenty-three inducible PHB lines were screened for PHB production in response to monochromatic red light. Dotted line represents the highest published cytosolic PHB yield in a plant (*n* = 2–3). Error bars represent SEM. (D) The relationship between PHB production and final plant size after 31 d of growth in the 23 screened lines. Average values for each line are plotted. Shaded area represents SEM. Pearson correlation coefficient (*r*) and *P* value (p) shown in top-right corner. (E) Western blot for the detection of β-ketothiolase (α-Myc), acetoacetyl-CoA reductase (α-PhbB), and PHB synthase (α-HA) in crude plant lysate of constitutive PHB lines. Loading is normalized based on amount of plant starting material. (F) Western blot for the detection of β-ketothiolase (α-Myc), acetoacetyl-CoA reductase (α-PhbB), and PHB synthase (α-HA) both post (+) and pre (-) induction in crude plant lysate of inducible PHB lines. Loading is normalized based on amount of plant starting material. (G) PHB levels expressed as total milligrams PHB per plant in both constitutive (#18, 25, 3) and inducible (#112, 88) lines grown from gemmae (*n* = 6). Error bars represent SEM. *** represents a *P* value < 0.001, ** a *P* value < 0.01, * a *P* value < 0.05, or n.s. a *P* value > 0.05 (Mann–Whitney *U*). (H) Final area of wild-type, constitutive PHB (lines #18, 25, 3), and inducible PHB (lines #112, 88) *Marchantia polymorpha* plants after growth for 31 d with or without induction according to the scheme shown in Figure 2D (*n* = 7–31). Error bars represent SEM. OE, overexpressor/transgenic lines; WT, wild type.
